# Supplementary material for: Actin filament assembly driven by distributive polymerases clustered on membrane surfaces
Source: bioRxiv. 2025 Oct 3:2024.11.26.625540. Preprint. [Version 2] doi: 10.1101/2024.11.26.625540 (PMC12621849; doi:10.1101/2024.11.26.625540)
Supplement: Supplement 1 [file NIHPP2024.11.26.625540v2-supplement-1.pdf]

bioRxiv preprint doi: https://doi.org/10.1101/2024.11.26.625540; this version posted October 4, 2025. The copyright holder for this preprint (which was not certified by peer review) is the author/funder, who has granted bioRxiv a license to display the preprint in perpetuity. It is made available under aCC-BY 4.0 International license.

Appendix A.
List of variables and parameters

λ_{p}- Persistence Length of an actin filament
L Length of actin filament
D_{pa} Diffusion coefficient of soluble profilin-actin complexes
D_{tip} Effective diffusion coefficient of thermally fluctuating actin filament tip
r_{disc} Root Mean Square (RMS) deflection of membrane-adjacent filament tip
r_{tip} Capture radius of the barbed end of an actin filament
r_{pol} Effective profilin-actin capture radius of a surface-bound polymerase
n_{pol} Number of polymerase molecules inside a membrane-interaction region
c_o Actin monomer concentration
$k_{on}c_o$ Rate of loading profilin-actin onto a surface-associated polymerase site
d_o Total surface density of profilin-actin binding sites ($/\mu m^2$)
d_{pa} Surface density of occupied profilin-actin binding sites ($/\mu m^2$)
R_{soln} Rate of soluble profilin-actin monomers encountering a filament tip
R_{2D} Rate of surface-associated profilin-actin encountering a nearby filament tip
R_{disc} Rate of soluble monomers encountering the membrane-interaction disc
R_{surf} Rate of surface-associated profilin-actin encountering a nearby filament tip
E_{soln} Filament elongation rate from soluble profilin-actin monomers
E_{surf} Filament elongation rate from surface-associated profilin-actin
E_{total} Filament elongation rate from soluble *and* surface-associated profilin-actin
f_{surf} Fraction of filamentous actin incorporated from surface polymerases
f_{OL} Fractional overlap of membrane-interaction regions of adjacent filaments
n_{eff} Effective number of competing filaments in a membrane interaction region
x_{ff} Distance between the equilibrium positions of two filaments tips

bioRxiv preprint doi: https://doi.org/10.1101/2024.11.26.625540; this version posted October 4, 2025. The copyright holder for this preprint (which was not certified by peer review) is the author/funder, who has granted bioRxiv a license to display the preprint in perpetuity. It is made available under aCC-BY 4.0 International license.

Appendix B.
Size of the *membrane interaction disc* traced by fluctuating filament tips

Filaments in a functional branched actin network generally range between 150-800 nm in length (Svitkina, 1999; Akin, 2008; Koestler, 2009), much shorter than their mechanical persistence length (λ_p). At these lengths, a filament behaves as a relatively stiff beam (Fig. B1). If we consider only small-amplitude, planar bends, deflection of the filament tip follows Hooke's Law, meaning that displacement from equilibrium (r) is directly proportional to applied force (F) :

$$F = \kappa r$$

Where the effective spring constant (κ) depends on the persistence length (λ_p) and actual filament length (L):

$$\kappa = k_B T \left(\frac{3\lambda_p}{L^3} \right)$$

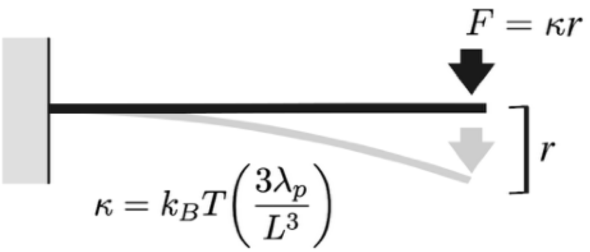

Figure B1. Small deflection bending of a stiff beam. The deflection is proportional to applied force, with an effective spring constant that depends on beam length and persistence length.

Where T is absolute temperature and k_B Boltzman's constant. From this, we can calculate the energy stored in the bent polymer:

$$E = \frac{1}{2}\kappa r^2 = k_B T \left(\frac{3\lambda_p}{2L^3} \right) r^2$$

If the average energy stored the bent polymer is $k_B T$, this yields:

$$k_B T = k_B T \left(\frac{3\lambda_p}{2L^3} \right) \langle r^2 \rangle$$

And the root-mean square (RMS) deviation of the tip is:

$$\sqrt{\langle r^2 \rangle} = \sqrt{\frac{2L^3}{3\lambda_p}}$$

Assuming an average filament length (L_{av}) of ~110 promoters (~300 nm), measured from the free barbed end to the network-anchored pointed end, the root-mean square (RMS) displacement of the tip will be ~45 nm.

bioRxiv preprint doi: https://doi.org/10.1101/2024.11.26.625540; this version posted October 4, 2025. The copyright holder for this preprint (which was not certified by peer review) is the author/funder, who has granted bioRxiv a license to display the preprint in perpetuity. It is made available under aCC-BY 4.0 International license.

Appendix C.
Analytical solution of a filament tip interacting with surface-bound polymerases via a simple, constrained random walk

Here we calculate the rate at which a filament tip undergoing a simple random walk, confined to a circular region of radius r_{disc} (calculated in Appendix B), interacts with randomly dispersed polymerase molecules. By "simple" random walk we mean that step direction does not depend on tip position (i.e. is not influenced by an elastic restoring force). We approximate the polymerase molecules as squares of length and width, a. (Figure C1). The number of actin-bound (occupied) polymerases inside the interaction disc is n_{op}, and the number of unoccupied polymerases is n_{up}. The fraction of occupied polymerases within the disc is, therefore,

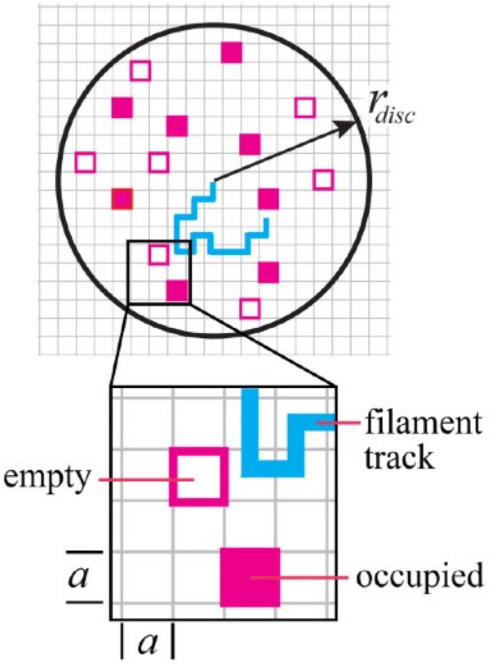

Figure C1. Top: filament tip (blue) doing a random walk on a circular membrane-interaction disc (radius - r_{disc}) filled with polymerase molecules (red squares). Open squares are unoccupied; filled squares are charged with profilin-actin. Bottom: zoom.

$$\rho = \frac{n_{op}}{n_{op} + n_{up}}$$

Surface-bound polymerases bind soluble profilin-actin complexes (concentration - c_o) at a rate of $k_{on}c_o$, where k_{on} is an effective rate constant whose value depends on the sizes of the molecules and the diffusion coefficient of the soluble profilin-actin. Under these conditions the total rate of profilin-actin uptake by unoccupied polymerases in the interaction disc is,

$$R_{uptake} = n_{up}\left(k_{on}c_o\right) = (1-\rho)\, d_o \pi r_{disc}^2 \left(k_{on}c_o\right)$$

bioRxiv preprint doi: https://doi.org/10.1101/2024.11.26.625540; this version posted October 4, 2025. The copyright holder for this preprint (which was not certified by peer review) is the author/funder, who has granted bioRxiv a license to display the preprint in perpetuity. It is made available under aCC-BY 4.0 International license.

where d_o is the surface density of WAVE/WASP-family polymerases. Note that, at steady state, the rate of surface-mediated filament elongation (R_{surf}) matches the total rate of profilin-actin uptake into the membrane interaction disc (R_{uptake}).

The motion of the filament tip is characterized by an effective diffusion coefficient, D_{tip} (Mogilner, 1996). We can approximate this by a discrete, two-dimensional random walk with step size a (the width of a polymerase molecule) and stepping rate k, given by

$$k = \frac{D_{tip}}{a^2}$$

The rate of filament elongation is now given by the stepping rate (k) multiplied by the probability that the destination square contains a profilin-actin-charged polymerase ($P_{occupied}$). To calculate the occupancy probability we divide the number of charged polymerases in the interaction disc by the total number of polymerases that can be packed into the disc

$$P_{occupied} = \frac{d_o \rho \pi r_{disc}^2}{\frac{\pi r_{disc}^2}{a^2}} = d_o a^2 \rho$$

And the rate of elongation becomes (**Eq. C1**)

$$R_{surf} = kP_{occupied} = kd_o a^2 \rho = \left(\frac{D_{tip}}{a^2}\right) d_o a^2 \rho = d_o D_{tip} \rho$$

At steady state, the rate of elongation matches the rate of profilin-actin uptake into the interaction disc, such that

$$d_o D_{tip} \rho = (1 - \rho)\, d_o \pi r_{disc}^2 \,(k_{on} c_o)$$

Solving for the fraction of occupied polymerases (ρ) yields

$$\rho = \frac{\pi \left(\frac{k_{on} c_o r_{disc}^2}{D_{tip}}\right)}{1 + \pi \left(\frac{k_{on} c_o r_{disc}^2}{D_{tip}}\right)}$$

Finally, substituting this expression for ρ into **Eq. C1** produces an equation for the elongation rate as a function of the soluble profilin-actin concentration (**Eq. C2**)

$$R_{surf} = d_o D_{tip} \left[\frac{\pi \left(\frac{k_{on} c_o r_{disc}^2}{D_{tip}}\right)}{1 + \pi \left(\frac{k_{on} c_o r_{disc}^2}{D_{tip}}\right)} \right]$$

bioRxiv preprint doi: https://doi.org/10.1101/2024.11.26.625540; this version posted October 4, 2025. The copyright holder for this preprint (which was not certified by peer review) is the author/funder, who has granted bioRxiv a license to display the preprint in perpetuity. It is made available under aCC-BY 4.0 International license.

Note that the term in parentheses is unitless. The value of r2disc/Dtip is proportional to the time required for the fluctuating tip to traverse the interaction disc while konco is the rate of profilin-actin delivery to a polymerase molecule. The product of these two terms, therefore, can be interpreted as the number profilin-actin complexes that can be delivered to each polymerase during one sweep of the filament across the disc.

bioRxiv preprint doi: https://doi.org/10.1101/2024.11.26.625540; this version posted October 4, 2025. The copyright holder for this preprint (which was not certified by peer review) is the author/funder, who has granted bioRxiv a license to display the preprint in perpetuity. It is made available under aCC-BY 4.0 International license.

Appendix D.
Numerical solution of Fick's equation in two dimensions with polymerase refilling from solution.

In this section we calculate a two-dimensional 'Smoluchowski limit' on the rate at which a growing filament tip interacts with profilin-actin-charged polymerase molecules on a membrane surface. We treat the filament tip as a circular hole whose radius (r_{tip}) represents the effective radius of interaction with polymerase-bound actin. For boundary conditions, we assume that the density of actin-bound polymerases is zero at the edge of the filament ($d_{pa}(r_{tip}) = 0$), and asymptotically approaches the total polymerase surface density (d_o) at long distances ($lim_{r\to\infty}[d_{pa}(r)] = d_o$). With these boundary conditions, we use Fick's Second Law of Diffusion to calculate the gradient of occupied polymerase molecules around the filament tip (Von Smoluchowski, 1917; Debye, 1942). Note that this approach is valid regardless of which species —filament or polymerase— undergoes diffusive motion (Debye, 1942).

If we neglect refilling of depleted polymerases by soluble profilin-actin complexes, and assume a radially symmetrical distribution of actin-bound polymerases around the filament tip, the steady-state form of Fick's Second Law in two dimensions is:

$$\frac{d}{dt}(d_{pa}) = D_{tip}\frac{1}{r}\left[\frac{\partial}{\partial r}\left(r\frac{\partial d_{pa}}{\partial r}\right)\right] = 0$$

Where D_{tip} is the diffusion coefficient of the filament tip skating across the membrane surface. Unlike the three-dimensional case, this partial differential equation has no solution that satisfies the given boundary conditions. If, however, we assume that unoccupied polymerase sites are refilled from a constant reservoir of soluble profilin-actin, the steady-state distribution of occupied polymerases must satisfy:

$$\frac{d}{dt}(d_{pa}) = D_{tip}\frac{1}{r}\left[\frac{\partial}{\partial r}\left(r\frac{\partial d_{pa}}{\partial r}\right)\right] + k_{on}c_o(d_o - d_{pa}) = 0 \qquad \textbf{Eqn. D1}$$

bioRxiv preprint doi: https://doi.org/10.1101/2024.11.26.625540; this version posted October 4, 2025. The copyright holder for this preprint (which was not certified by peer review) is the author/funder, who has granted bioRxiv a license to display the preprint in perpetuity. It is made available under aCC-BY 4.0 International license.

The additional term in this equation represents the rate at which unoccupied polymerases are recharged from solution. This depends on the density of unoccupied polymerases (d_o-d_{pa}), the solution concentration of profilin-actin complexes (c_o), and a rate constant (k_{on}) for binding of soluble proteins to membrane-associated sites (Berg, 1977).

To solve this equation for the surface density of loaded polymerases (d_{pa}) we first make the following three substitutions:

$$\rho = \left(\frac{r}{r_{tip}}\right) \qquad d = (d_{pa} - d_o) \qquad \alpha^2 = \left(\frac{k_{on} c_o r_{tip}^2}{D_{tip}}\right)$$

With these substitutions, we can rearrange **Eqn. D1** into the following form:

$$\rho^2 \frac{\partial^2}{\partial \rho^2} d + \rho \frac{\partial}{\partial \rho} d - \alpha^2 \rho^2 d = 0 \qquad \textbf{Eqn. D2}$$

This rearranged equation has the form of a modified Bessel's Equation of order zero, whose solution is given by a linear combination of the zero-order modified Bessel functions, I_o and K_o.

$$d(\rho) = C_1 I_o(\alpha\rho) + C_2 K_o(\alpha\rho)$$

Where C_1 and C_2 are constants. We determine the values of C_1 and C_2 by applying the boundary conditions. The function I_o grows without bound, so the condition that d is bounded requires C_1=0. When $r = r_{tip}$, the substituted parameter $\gamma = 1$. The boundary condition that $d_{pa}(r_{tip}) = 0$, therefore, implies that

$$d_{pa}|_{\gamma=1} = 0 \implies 0 = C_2 K_o(\alpha) + d_o$$

And

$$C_2 = -\frac{d_o}{K_o \alpha}$$

Using this value for C_2 and substituting for d and ρ, we obtain a closed-form solution for the steady-state distribution of actin-charged polymerases

$$d_{pa}(r) = d_o \left[1 - \frac{K_o(\alpha r / r_{tip})}{K_o(\alpha)}\right] \qquad \textbf{Eqn. D3}$$

bioRxiv preprint doi: https://doi.org/10.1101/2024.11.26.625540; this version posted October 4, 2025. The copyright holder for this preprint (which was not certified by peer review) is the author/funder, who has granted bioRxiv a license to display the preprint in perpetuity. It is made available under aCC-BY 4.0 International license.

We can use this formula, together with Fick's first law of diffusion, to compute the rate (R_{2D}) at which the filament tip interacts with surface-associated actin monomers. We simply multiply the diffusion coefficient of the filament tip (D_{tip}), the density gradient at the filament edge ($\partial d_{pa}/\partial r\,|_{r=r_{tip}}$), and the circumference of the monomer binding site on the end of the filament ($2\pi r_{tip}$).

$$R_{2D} = 2\pi r_{tip}\left(D_{tip}\,\frac{\partial d_{pa}}{\partial r}\bigg|_{r=r_{tip}}\right)$$

Substituting **Eqn. D3** into the above expression yields

$$R_{2D} = 2\pi r_{tip} D_{tip}\,\frac{\partial d_{pa}}{\partial r}\left[d_o\left(1-\frac{K_o(\alpha r/r_{tip})}{K_o(\alpha)}\right)\right]\bigg|_{r=r_{tip}}$$

Which becomes

$$R_{2D} = 2\pi D_{tip} d_o \alpha\frac{K_1(\alpha)}{K_o(\alpha)} \qquad \textbf{Eqn. D3}$$

Where K_1 is a modified Bessel function of order one. Note that the dimensionless parameter α^2 resembles the previously defined parameter, δ_1

$$\delta_1 = k_{on}c_o\left(\frac{r_{disc}^2}{D_{tip}}\right) \qquad \alpha^2 = k_{on}c_o\left(\frac{r_{tip}^2}{D_{tip}}\right)$$

Because of this similarity, we define a new parameter, $\delta_2 = \alpha^2$. A physical interpretation of δ_1 is that it represents the number of times one polymerase molecule can be loaded from solution with profilin-actin during the time it takes the filament tip to traverse the entire membrane interaction disc. Similarly, δ_2 can be understood as the number of profilin-actin complexes

Table C1. Parameter ranges and exponents

**Parameter**	**Range**	**Exponent**
c_o	10-400 µM	0.125
r_{tip},	0.5-8 nm	0.25
D_{tip}	1-16 µm²/s	0.875
d_o	2000-6000/µm²	1.0

bioRxiv preprint doi: https://doi.org/10.1101/2024.11.26.625540; this version posted October 4, 2025. The copyright holder for this preprint (which was not certified by peer review) is the author/funder, who has granted bioRxiv a license to display the preprint in perpetuity. It is made available under aCC-BY 4.0 International license.

that can bind a polymerase molecule during the course of one filament-polymerase interaction. It makes intuitive sense that the rate membrane-dependent filament elongation would depend on these parameters.

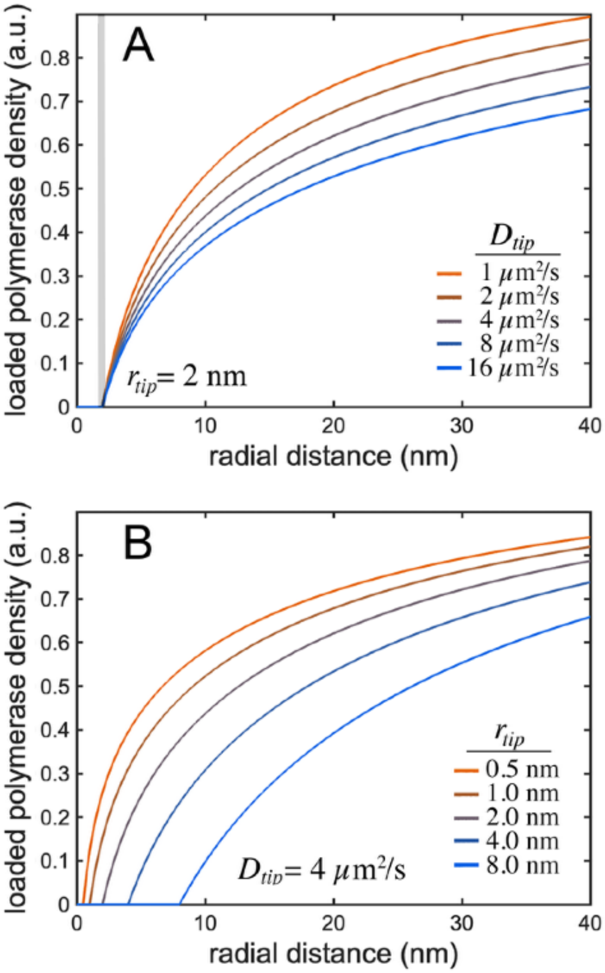

Figure D1. Steady-state distributions of actin-charged polymerases around an absorbing filament tip, determined by numerical simulation with various parameter values: (A) varying diffusion coefficients and (B) varying capture radii of the filament tip. For all conditions, c_o=100μM.

To validate our analytical solution and find a simpler, approximate expression for R_{2D}, we performed a numerical simulation of two-dimensional diffusion of ligands into an absorbing disc located at the origin. Briefly, we modeled diffusion in radial coordinates on a set of concentric rings, each with a fixed width (δ). Given values for δ and filament tip diffusion coefficient (D_{tip}) we chose a time step (τ) such that $\tau = \delta^2/D_{tip}$. We set up an initial, uniform distribution of loaded polymerases, with $d_{pa} = d_o$ for all $r > r_{tip}$, and stable boundary conditions of $d_{pa} = 0$ at $r = r_{tip}$ and $d_{pa} = d_o$ at $r > 1000\delta$. At each time step, τ, we assumed that the molecules in each concentric ring move inward or outward with the ratio of in-to-out determined by the areas of the adjacent rings. We then calculated the number of profilin-actin complexes recruited to each ring from solution by multiplying the area of the ring by the density of unoccupied polymerases, the solution concentration of profilin-actin, the rate constant for polymerase charging and the time increment: $k_{on}c_o(d_o\text{-}d_{pa})\tau$. We obtained the steady-state distribution

bioRxiv preprint doi: https://doi.org/10.1101/2024.11.26.625540; this version posted October 4, 2025. The copyright holder for this preprint (which was not certified by peer review) is the author/funder, who has granted bioRxiv a license to display the preprint in perpetuity. It is made available under aCC-BY 4.0 International license.

of actin-charged polymerases by iterating time steps until the change in distribution became negligible. We validated this approach by simulating diffusion into a three-dimensional, spherical absorber and comparing the results to von Smoluchowski (1917).

Our simulations produced stable distributions of actin-charged polymerases around the filament tip that matched the distributions calculated from our analytical solution, **Eqn. D4** (Figure D1). We used these distributions to calculate the flux of actin into the filament (R_{2D}) at different values of the relevant parameters ($k_{on}c_o$, r_{tip}, D_{tip}, and d_o).

Across physiologically relevant values of each parameter (Table D1), the interaction rate was well approximated as a simple power law (Figure D2, Table D1). Combining the dependences on all of the parameters into a single equation yields:

$$R_{2D} \approx \left[2.6 D_{tip}^{0.875} \left(r_{tip}^2 k_{on} c_o\right)^{0.125}\right] d_o = k'_+ d_o \qquad \textbf{Eqn. D2}$$

The above equation can be rearranged into a slightly more interpretable form:

$$R_{2D} \approx \left[2.6 D_{tip} \left(\frac{r_{tip}^2 k_{on} c_o}{D_{tip}}\right)^{0.125}\right] d_o = k' d_o$$

The product of diffusion coefficient and surface actin density ($D_{tip}d_o$) is proportional to the rate of collision between a polymerase and the filament tip, while the unitless term in parentheses (defined above as δ_2) can be interpreted as the number of times a polymerase can be loaded with profilin-actin from solution during a single collision event. The term in square brackets has the form of a rate constant (k').

bioRxiv preprint doi: https://doi.org/10.1101/2024.11.26.625540; this version posted October 4, 2025. The copyright holder for this preprint (which was not certified by peer review) is the author/funder, who has granted bioRxiv a license to display the preprint in perpetuity. It is made available under aCC-BY 4.0 International license.

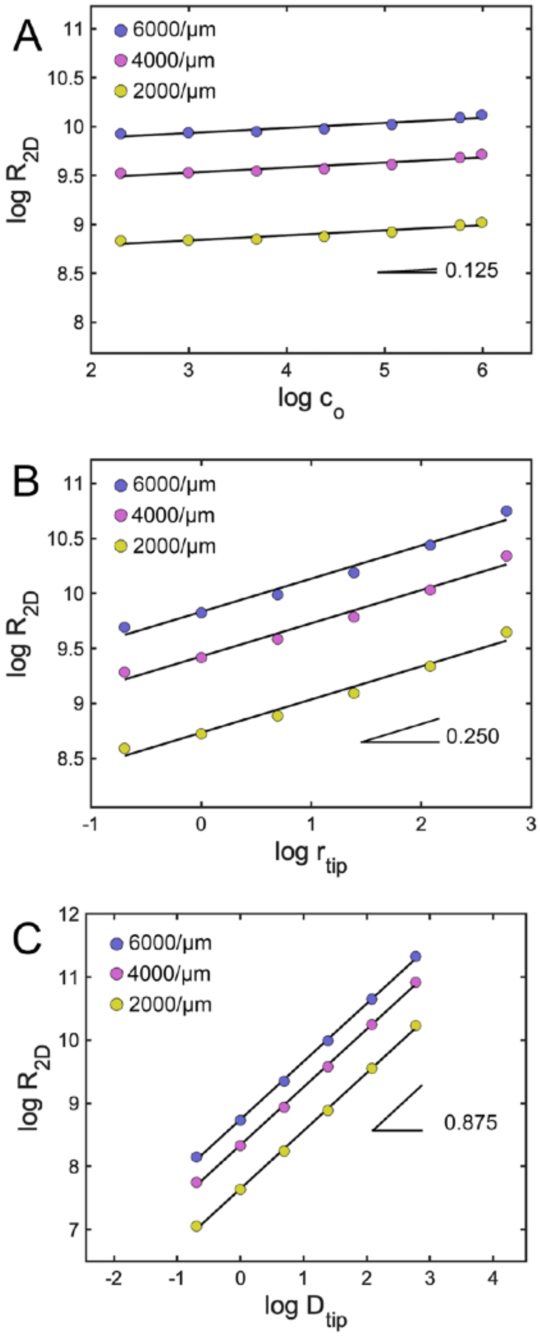

Figure D2. Log-log plots of 2D filament encounter rate (R_{2D}) versus key parameter values. (A) R_{2D} vs. soluble profilin-actin concentration (c_o). Slope: 0.125. (B) R_{2D} vs. filament capture radius (r_{tip}). Slope: 0.25. (C) R_{2D} vs. tip diffusion coefficient (D_{tip}). Slope: 0.875. Baseline parameter values: c_o=100 µM; r_{tip}=2 nm; D_{tip}=4 µm²/sec.
